# Successful public and patient involvement in bowel cancer research using linked administrative health data.

**DOI:** 10.23889/ijpds.v7i3.2014

**Published:** 2022-08-25

**Authors:** Elizabeth Lemmon, Emily Connearn, Peter Hall, Eva Morris, UK Colorectal Intelligence Hub And Yorkshire Cancer Research Bowel Cancer Improvement Programme Public-Patient Group

**Affiliations:** 1 University of Edinburgh; 2 Edinburgh Health Economics; 3 University of Leeds; 4 University of Oxford

This poster aims to highlight the impact of a public/patient group (PPG) involved in two large programmes that are researching bowel cancer using routine, linked administrative datasets in England and Scotland.

The PPG consists of ten independent patients or carers who have lived, or shared, experiences of bowel cancer. Members are involved in the full life cycle of individual research projects from project development, to research in progress and onto dissemination of the work once published. They are actively involved in shaping the strategic direction of the programmes and are a key part of the data access process. The group meets once a month and acts as a ‘critical friend’ to the researchers.

The PPG have produced over 18 plain language summaries to ensure accessibility of the programmes’ research to everyone and that it reaches those directly affected by bowel cancer. They have produced four publications including one Editorial, one Opinion piece and two co-authored manuscripts. The group have also co-developed patient information sheets and consent forms with clinicians that are now used in various cancer centres across Yorkshire. Furthermore, one of the PPG members co-presenting a webinar, which highlighted their involvement in developing a unique linked bowel cancer dataset to an international audience of over 100 people.

The PPG has demonstrated that involving the public and patients in the development of data linkage research is hugely beneficial. Their experience and knowledge shapes research that ultimately improves care. The success of our PPG is an exemplar for embedding patients and public involvement in research.

